# 3-[(Furan-2-yl)carbon­yl]-1-(pyrimi­din-2-yl)thio­urea

**DOI:** 10.1107/S1600536812044029

**Published:** 2012-11-07

**Authors:** Durga P. Singh, Seema Pratap, Sushil K. Gupta, Ray J. Butcher

**Affiliations:** a Department of Chemistry, M.M.V., Banaras Hindu University, Varanasi 221 005, India; b School of Studies in Chemistry, Jiwaji University, Gwalior 474 011, India; c Department of Chemistry, Howard University, 525 College Street NW, Washington, DC 20059, USA

## Abstract

The title compound, C_10_H_8_N_4_O_2_S, was synthesized from furoyl isothio­cynate and 2-amino­pyrimidine in dry acetone. The two N—H groups are in an *anti* conformation with respect to each other and one N—H group is *anti* to the C=S group while the other is *syn*. The amide C=S and the C=O groups are *syn* to each other. The mean plane of the central thio­urea fragment forms dihedral angles of 13.50 (14) and 5.03 (11)° with the furan and pyrimidine rings, respectively. The dihedral angle between the furan and pyrimidine rings is 18.43 (10)°. The mol­ecular conformation is stabilized by an intra­molecular N—H⋯N hydrogen bond generating an *S*(6) ring motif. In the crystal, mol­ecules are linked by pairs of N—H⋯N and weak C—H⋯S hydrogen bonds to form inversion dimers.

## Related literature

For a general background to the biological activity of thio­urea, see: Koketsu & Ishihara (2006 ▶). For heterocyclic derivatives, metal complexes and mol­ecular electronics, see: Zeng *et al.* (2003 ▶); D’hooghe *et al.* (2005 ▶); Aly *et al.* (2007 ▶); Duque *et al.* (2009 ▶). For related structures, see: Singh *et al.* (2012 ▶); Koch (2001 ▶); Hassan *et al.* (2007 ▶); Pérez *et al.* (2008 ▶); Yan & Xue (2008 ▶).
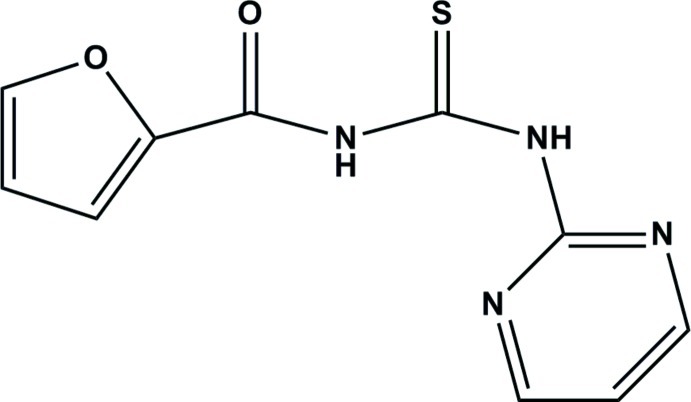


## Experimental

### 

#### Crystal data

C_10_H_8_N_4_O_2_S
*M* *_r_* = 248.26
Monoclinic, 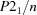

*a* = 5.6962 (2) Å
*b* = 21.0530 (7) Å
*c* = 8.7901 (3) Å
β = 95.559 (3)°
*V* = 1049.17 (6) Å^3^
*Z* = 4
Cu *K*α radiation
μ = 2.74 mm^−1^
*T* = 123 K
0.40 × 0.22 × 0.11 mm

#### Data collection

Agilent Xcalibur (Ruby, Gemini) diffractometer
Absorption correction: multi-scan (*CrysAlis PRO*; Agilent, 2011 ▶) *T*_min_ = 0.421, *T*_max_ = 1.000
6957 measured reflections
2028 independent reflections
1951 reflections with *I* > 2σ(*I*)
*R*_int_ = 0.028

#### Refinement

*R*[*F* ^2^ > 2σ(*F* ^2^)] = 0.038
*wR*(*F* ^2^) = 0.108
*S* = 1.04
2028 reflections
154 parameters
H-atom parameters constrained
Δρ_max_ = 0.46 e Å^−3^
Δρ_min_ = −0.21 e Å^−3^

### 

Data collection: *CrysAlis PRO* (Agilent, 2011 ▶); cell refinement: *CrysAlis PRO*; data reduction: *CrysAlis PRO*; program(s) used to solve structure: *SHELXS97* (Sheldrick, 2008 ▶); program(s) used to refine structure: *SHELXL97* (Sheldrick, 2008 ▶); molecular graphics: *SHELXTL* (Sheldrick, 2008 ▶); software used to prepare material for publication: *SHELXTL*.

## Supplementary Material

Crystal structure: contains datablock(s) global, I. DOI: 10.1107/S1600536812044029/lh5548sup1.cif

Structure factors: contains datablock(s) I. DOI: 10.1107/S1600536812044029/lh5548Isup2.hkl

Supplementary material file. DOI: 10.1107/S1600536812044029/lh5548Isup3.cml

Additional supplementary materials:  crystallographic information; 3D view; checkCIF report

## Figures and Tables

**Table 1:** Hydrogen-bond geometry (Å, °)

*D*—H⋯*A*	*D*—H	H⋯*A*	*D*⋯*A*	*D*—H⋯*A*
N1—H1*B*⋯N3	0.86	1.89	2.6240 (18)	142
N2—H2*B*⋯N4^i^	0.86	2.21	3.0726 (19)	175
C10—H10*A*⋯S1^i^	0.93	2.76	3.5536 (17)	144

Symmetry code: (i) 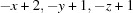.

**Fig. 1.**
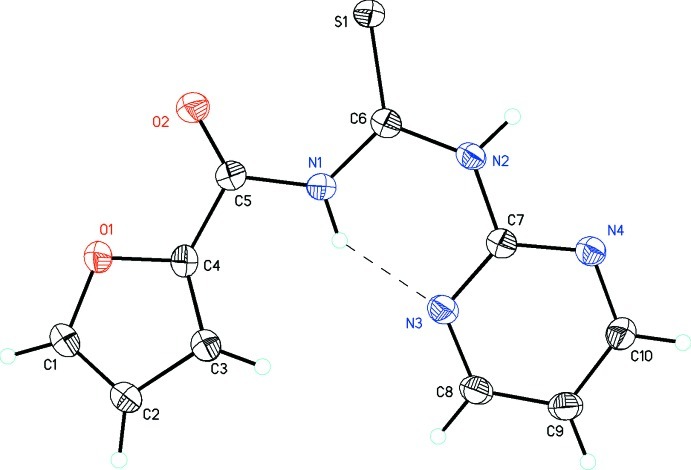
The molecular structure of the title compound showing 30% probability displacement ellipsoids. Dashed lines indicate an intramolecular N—H···N hydrogen bond.

**Fig. 2.**
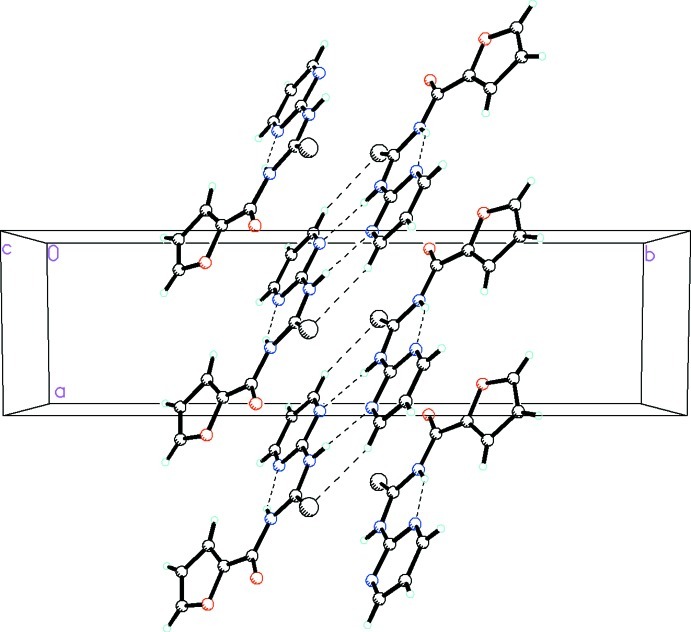
Crystal packing for the title compound viewed along the c axis. Dashed lines indicate intermolecular N—H···N and C—H···S hydrogen bonds.

## supplementary crystallographic information

### Comment

Thiourea and its derivatives are known to exhibit a wide variety of biological activities (Koketsu & Ishihara, 2006). These are also widely used as precursors or intermediates towards the syntheisis of a variety of heterocyclic compounds (Zeng *et al.*, 2003; D'hooghe, *et al.*, 2005). In addition, aroylthioureas have applications in metal complexes and molecular electronics (Aly *et al.*, 2007; Duque *et al.*, 2009). The structure of a related compound was recently published (Yan & Xue, 2008) in which the molecule showed excellent herbicidal activity.

In view of the biological importance of thiourea and its furoic acid derviatives, the structure of the title compound was determined. In the title compound (Fig. 1), the conformation of the two N—H bonds are *anti* to each other, and one of them is *anti* to the C═S and the other is *syn* in the urea moiety. Furthermore, the amide C═S and the C═O groups are *syn* to each other, similar to the *syn* conformation observed in 1-furoyl-3-methyl-3-phenylthiourea (Pérez *et al.*, 2008) and in *N*-(2-furoyl)-*N*^'^(6-methyl-2-pyridyl)thiourea (Hassan *et al.*, 2007). The bond lengths and angles in the title compound are comparable to other thiourea derivatives (Koch 2001; Pérez *et al.*, 2008; Singh *et al.*, 2012). The C6—S1 and C5—O2 bonds show typical double-bond character. However, the C—N bond lengths, C5—N1, C6—N1, C6—N2 are shorter than the normal C—N single-bond length of about 1.48 Å. These results can be explained by the existence of resonance in this part of the molecule. The central thiourea fragment (O2/C5/N1/C6/N2) makes dihedral angle of 13.50 (14)° with furan ring (O1/C1/C2/C3/C4)and 5.03 (11)° with pyrimidine ring (C7/N3/C8/C9/C10/N4), respectively. The dihedral angle between the mean planes of the furan and pyrimidine rings is 18.43 (10)°. The moleculer geometry is stabilized by an intramolecular N—H···N hydrogen bond generating an S(6) ring motif. In the crystal, molecules are linked by pairs of N—H···N and weak C—H···S hydrogen bonds (Table 1) forming centrosymmetric dimers (Fig. 2).

### Experimental

A solution of 2-thiophenecarbonyl chloride (0.01 mol) in anhydrous acetone (80 ml) was added dropwise to a suspension of ammonium thiocyanate (0.01 mol) in anhydrous acetone (50 ml) and the reaction mixture was refluxed for 50 minutes. After cooling to room temperature, a solution of 4-chloroaniline (0.01 mol) in dry acetone (25 ml) was added and the resulting mixture refluxed for 2 h. The reaction mixture was poured into five times its volume of cold water, upon which the thiourea precipitated. The resulting solide product was crystallized from acetone yielding yellow colour X-ray quality single crystals. Yield: 80%; M.P.: 455 - 456 K). Anal. Calc. for C_10_H_8_N_4_O_2_S (%): C, 48.38; H,3.25; N, 22.57. Found: C, 48.49; H, 3.28; N, 22.50.

### Refinement

All H atoms were placed in calculated positions and refined using a riding-model approximation with C—H = 0.93 Å, N—H = 0.86Å and *U*_iso_(H) = 1.2*U*_eq_(C,N).

### Crystal data

**Table:**

C_10_H_8_N_4_O_2_S	*F*(000) = 512
*M**_r_* = 248.26	*D*_x_ = 1.572 Mg m^−^^3^
Monoclinic, *P*2_1_/*n*	Cu *K*α radiation, λ = 1.54178 Å
Hall symbol: -P 2yn	Cell parameters from 4415 reflections
*a* = 5.6962 (2) Å	θ = 4.2–72.7°
*b* = 21.0530 (7) Å	µ = 2.74 mm^−^^1^
*c* = 8.7901 (3) Å	*T* = 123 K
β = 95.559 (3)°	Plate, colorless
*V* = 1049.17 (6) Å^3^	0.40 × 0.22 × 0.11 mm
*Z* = 4	

### Data collection

**Table:**

Agilent Xcalibur (Ruby, Gemini) diffractometer	2028 independent reflections
Radiation source: Enhance (Cu) X-ray Source	1951 reflections with *I* > 2σ(*I*)
Graphite monochromator	*R*_int_ = 0.028
Detector resolution: 10.5081 pixels mm^-1^	θ_max_ = 72.8°, θ_min_ = 4.2°
ω scans	*h* = −6→5
Absorption correction: multi-scan (*CrysAlis PRO*; Agilent, 2011)	*k* = −25→25
*T*_min_ = 0.421, *T*_max_ = 1.000	*l* = −10→9
6957 measured reflections	

### Refinement

**Table:**

Refinement on *F*^2^	Primary atom site location: structure-invariant direct methods
Least-squares matrix: full	Secondary atom site location: difference Fourier map
*R*[*F*^2^ > 2σ(*F*^2^)] = 0.038	Hydrogen site location: inferred from neighbouring sites
*wR*(*F*^2^) = 0.108	H-atom parameters constrained
*S* = 1.04	*w* = 1/[σ^2^(*F*_o_^2^) + (0.0735*P*)^2^ + 0.4136*P*] where *P* = (*F*_o_^2^ + 2*F*_c_^2^)/3
2028 reflections	(Δ/σ)_max_ = 0.001
154 parameters	Δρ_max_ = 0.46 e Å^−^^3^
0 restraints	Δρ_min_ = −0.21 e Å^−^^3^

### Special details

**Table:**

Experimental. FT IR (selected, KBr, cm^-1^):3424, 3207 [ν(N – H)]; 1712 [amide-I,*C*═O]; 1586, 1556 [ν(C═C)]; 1505[thioureido-I], 1327 [thioureido-II], 1177 [thioureido-III], 763 [thioureido-IV]. ^1^H NMR (300 MHz, dmso-d_6_): δ 14.08 (s, 1H, H-bonded N–H); 11.90 (s, 1H,free N–H); 8.80 (d,*J* = 7.1 Hz, 2H, pyrimidine CH); 8.05 (d, *J* = 7.5 Hz, 1H, furan CH); 7.50(d,*J* = 7.8 Hz, 1H, pyrimidine CH);7.30 (t, *J*_1_(H,H) = 6.8 Hz,*J*_2_(H,H) = 7.1 Hz, 1H, pyrimidine CH);6.76 (t, *J*(H,H) = 7.8 Hz, 1H, furan CH). ^13^C NMR (75 MHz,dmso-d_6_): δ 177.4, 158.5, 157.1, 155.4, 147.6, 146.4, 117.6, 117.1, 112.9.
Geometry. All e.s.d.'s (except the e.s.d. in the dihedral angle between two l.s. planes) are estimated using the full covariance matrix. The cell e.s.d.'s are taken into account individually in the estimation of e.s.d.'s in distances, angles and torsion angles; correlations between e.s.d.'s in cell parameters are only used when they are defined by crystal symmetry. An approximate (isotropic) treatment of cell e.s.d.'s is used for estimating e.s.d.'s involving l.s. planes.
Refinement. Refinement of *F*^2^ against ALL reflections. The weighted *R*-factor *wR* and goodness of fit *S* are based on *F*^2^, conventional *R*-factors *R* are based on *F*, with *F* set to zero for negative *F*^2^. The threshold expression of *F*^2^ > σ(*F*^2^) is used only for calculating *R*-factors(gt) *etc*. and is not relevant to the choice of reflections for refinement. *R*-factors based on *F*^2^ are statistically about twice as large as those based on *F*, and *R*- factors based on ALL data will be even larger.

### Fractional atomic coordinates and isotropic or equivalent isotropic displacement parameters (Å^2^)

**Table:**

	*x*	*y*	*z*	*U*_iso_*/*U*_eq_	
S1	0.47026 (7)	0.555126 (18)	0.19269 (4)	0.02308 (17)	
O1	−0.1466 (2)	0.71757 (6)	0.45239 (14)	0.0270 (3)	
O2	0.0499 (2)	0.63600 (6)	0.26198 (14)	0.0283 (3)	
N1	0.3619 (2)	0.61674 (6)	0.44577 (15)	0.0214 (3)	
H1B	0.4047	0.6248	0.5403	0.026*	
N2	0.7006 (2)	0.55592 (6)	0.46685 (16)	0.0201 (3)	
H2B	0.7901	0.5300	0.4235	0.024*	
N3	0.6446 (2)	0.60768 (6)	0.69888 (15)	0.0234 (3)	
N4	0.9782 (2)	0.54126 (7)	0.67136 (15)	0.0216 (3)	
C1	−0.1833 (3)	0.76232 (8)	0.5596 (2)	0.0285 (4)	
H1A	−0.3194	0.7867	0.5597	0.034*	
C2	0.0017 (3)	0.76645 (8)	0.6641 (2)	0.0304 (4)	
H2A	0.0183	0.7937	0.7478	0.036*	
C3	0.1690 (3)	0.72089 (8)	0.6224 (2)	0.0288 (4)	
H3A	0.3162	0.7124	0.6737	0.035*	
C4	0.0718 (3)	0.69251 (7)	0.49378 (18)	0.0214 (3)	
C5	0.1539 (3)	0.64564 (7)	0.38553 (18)	0.0204 (3)	
C6	0.5082 (3)	0.57705 (7)	0.37439 (17)	0.0192 (3)	
C7	0.7750 (3)	0.56938 (7)	0.61922 (18)	0.0196 (3)	
C8	0.7219 (3)	0.61687 (8)	0.84626 (19)	0.0257 (4)	
H8A	0.6344	0.6426	0.9055	0.031*	
C9	0.9258 (3)	0.58957 (8)	0.91292 (19)	0.0261 (4)	
H9A	0.9775	0.5958	1.0154	0.031*	
C10	1.0498 (3)	0.55213 (8)	0.81814 (19)	0.0250 (4)	
H10A	1.1902	0.5336	0.8591	0.030*	

### Atomic displacement parameters (Å^2^)

**Table:**

	*U*^11^	*U*^22^	*U*^33^	*U*^12^	*U*^13^	*U*^23^
S1	0.0259 (3)	0.0236 (3)	0.0195 (3)	0.00267 (13)	0.00100 (17)	−0.00364 (13)
O1	0.0233 (6)	0.0286 (6)	0.0280 (6)	0.0067 (5)	−0.0032 (5)	−0.0046 (5)
O2	0.0252 (6)	0.0347 (7)	0.0245 (6)	0.0026 (5)	−0.0006 (5)	−0.0060 (5)
N1	0.0242 (7)	0.0215 (6)	0.0182 (6)	0.0022 (5)	0.0012 (5)	−0.0023 (5)
N2	0.0232 (7)	0.0180 (6)	0.0195 (7)	0.0021 (5)	0.0041 (5)	−0.0022 (5)
N3	0.0273 (7)	0.0210 (6)	0.0218 (7)	0.0036 (5)	0.0024 (5)	−0.0017 (5)
N4	0.0224 (7)	0.0207 (6)	0.0217 (7)	0.0005 (5)	0.0026 (5)	−0.0017 (5)
C1	0.0292 (9)	0.0246 (8)	0.0315 (9)	0.0081 (6)	0.0023 (7)	−0.0038 (6)
C2	0.0315 (9)	0.0282 (9)	0.0304 (9)	0.0086 (7)	−0.0025 (7)	−0.0094 (7)
C3	0.0264 (8)	0.0286 (8)	0.0303 (9)	0.0072 (7)	−0.0030 (7)	−0.0073 (7)
C4	0.0198 (7)	0.0201 (7)	0.0240 (8)	0.0013 (6)	0.0010 (6)	0.0024 (6)
C5	0.0212 (7)	0.0194 (7)	0.0206 (7)	−0.0026 (6)	0.0023 (6)	0.0006 (6)
C6	0.0219 (7)	0.0152 (7)	0.0210 (7)	−0.0023 (5)	0.0042 (6)	0.0003 (5)
C7	0.0224 (8)	0.0163 (7)	0.0205 (8)	−0.0012 (6)	0.0037 (6)	0.0014 (6)
C8	0.0316 (9)	0.0240 (8)	0.0218 (8)	0.0038 (6)	0.0041 (6)	−0.0034 (6)
C9	0.0323 (9)	0.0253 (8)	0.0202 (8)	0.0011 (6)	0.0000 (6)	−0.0034 (6)
C10	0.0241 (8)	0.0258 (9)	0.0245 (9)	0.0022 (6)	−0.0011 (7)	0.0002 (6)

### Geometric parameters (Å, º)

**Table:**

S1—C6	1.6565 (15)	N4—C7	1.340 (2)
O1—C1	1.363 (2)	C1—C2	1.332 (3)
O1—C4	1.3679 (19)	C1—H1A	0.9300
O2—C5	1.203 (2)	C2—C3	1.425 (2)
N1—C6	1.3739 (19)	C2—H2A	0.9300
N1—C5	1.390 (2)	C3—C4	1.349 (2)
N1—H1B	0.8600	C3—H3A	0.9300
N2—C6	1.373 (2)	C4—C5	1.478 (2)
N2—C7	1.394 (2)	C8—C9	1.375 (2)
N2—H2B	0.8600	C8—H8A	0.9300
N3—C7	1.339 (2)	C9—C10	1.389 (2)
N3—C8	1.341 (2)	C9—H9A	0.9300
N4—C10	1.335 (2)	C10—H10A	0.9300
			
C1—O1—C4	106.22 (13)	O1—C4—C5	115.00 (14)
C6—N1—C5	128.64 (13)	O2—C5—N1	126.67 (15)
C6—N1—H1B	115.7	O2—C5—C4	122.34 (15)
C5—N1—H1B	115.7	N1—C5—C4	110.98 (13)
C6—N2—C7	130.81 (13)	N2—C6—N1	114.28 (13)
C6—N2—H2B	114.6	N2—C6—S1	120.16 (11)
C7—N2—H2B	114.6	N1—C6—S1	125.56 (12)
C7—N3—C8	116.43 (14)	N3—C7—N4	126.26 (14)
C10—N4—C7	115.33 (14)	N3—C7—N2	119.48 (14)
C2—C1—O1	110.97 (15)	N4—C7—N2	114.26 (13)
C2—C1—H1A	124.5	N3—C8—C9	122.41 (15)
O1—C1—H1A	124.5	N3—C8—H8A	118.8
C1—C2—C3	106.41 (15)	C9—C8—H8A	118.8
C1—C2—H2A	126.8	C8—C9—C10	116.07 (15)
C3—C2—H2A	126.8	C8—C9—H9A	122.0
C4—C3—C2	106.46 (15)	C10—C9—H9A	122.0
C4—C3—H3A	126.8	N4—C10—C9	123.46 (15)
C2—C3—H3A	126.8	N4—C10—H10A	118.3
C3—C4—O1	109.94 (14)	C9—C10—H10A	118.3
C3—C4—C5	134.87 (15)		
			
C4—O1—C1—C2	0.5 (2)	C7—N2—C6—S1	177.23 (12)
O1—C1—C2—C3	−0.5 (2)	C5—N1—C6—N2	−179.49 (14)
C1—C2—C3—C4	0.3 (2)	C5—N1—C6—S1	1.3 (2)
C2—C3—C4—O1	0.0 (2)	C8—N3—C7—N4	−2.3 (2)
C2—C3—C4—C5	174.44 (18)	C8—N3—C7—N2	177.72 (14)
C1—O1—C4—C3	−0.26 (18)	C10—N4—C7—N3	1.7 (2)
C1—O1—C4—C5	−175.93 (14)	C10—N4—C7—N2	−178.32 (13)
C6—N1—C5—O2	8.1 (3)	C6—N2—C7—N3	2.0 (2)
C6—N1—C5—C4	−171.00 (14)	C6—N2—C7—N4	−177.98 (14)
C3—C4—C5—O2	−165.63 (19)	C7—N3—C8—C9	1.0 (2)
O1—C4—C5—O2	8.6 (2)	N3—C8—C9—C10	0.6 (2)
C3—C4—C5—N1	13.5 (3)	C7—N4—C10—C9	0.2 (2)
O1—C4—C5—N1	−172.23 (12)	C8—C9—C10—N4	−1.3 (2)
C7—N2—C6—N1	−2.0 (2)		

### Hydrogen-bond geometry (Å, º)

**Table:**

*D*—H···*A*	*D*—H	H···*A*	*D*···*A*	*D*—H···*A*
N1—H1*B*···N3	0.86	1.89	2.6240 (18)	142
N2—H2*B*···N4^i^	0.86	2.21	3.0726 (19)	175
C10—H10*A*···S1^i^	0.93	2.76	3.5536 (17)	144

Symmetry code: (i) −*x*+2, −*y*+1, −*z*+1.
